# Effect of salt leaching on micromechanical behaviour and structure of Hachirogata clay

**DOI:** 10.1038/s41598-025-34305-8

**Published:** 2026-01-04

**Authors:** Motohei Kanayama, Momoe Takahashi, Kiyohito Yamamoto

**Affiliations:** 1 Department of Agro‑Environmental Sciences, Faculty of Agriculture, Kyushu University, 744 Motooka, Nishi‑ku, Fukuoka, Japan; 2 Kanto Branch, Kajima Corporation, 2‑118 Daimoncho, Omiya‑ku, Saitama, Japan; 3 Faculty of Agriculture, Iwate University, 3-18-8 Ueda, Morioka, Japan

**Keywords:** Marine clay, Salt leaching, Micro-indentation test, Electrical conductivity, Cohesion, Microscopic structure, Engineering, Environmental sciences, Materials science, Natural hazards, Solid Earth sciences

## Abstract

In the Hachirogata reclaimed area, Minamiakita County, Akita Prefecture, Japan, one of the leading agricultural production areas in the country, the agricultural production foundation and facilities are deteriorating significantly, and problems specific to low-lying reclaimed farmland, such as deterioration of water quality and differential settlement of structures, are occurring. This study investigates the effects of salt leaching on the micromechanical behaviour of highly active Hachirogata clay. A leached sample was artificially prepared using a boring sample collected from the Hachirogata reclaimed land, and a micro-indentation test was performed on both non-leached and leached samples. The results of the indentation test confirmed that the electrical conductivity (EC) of Hachirogata clay was highly correlated with cohesion, c, and that the c value dropped sharply when the EC fell below a certain threshold. Therefore, salt leaching leads to a decrease in the cohesion of clay particles by reducing the number of ions in the clay, making the clay easier to deform under load. From the results of this study, it was inferred that localised salt leaching, which changes the micromechanical behaviour of clay, is one of the factors causing ground differential settlement or slip failure. It is expected that such ground problems could be mitigated by identifying locations with low EC values, that is, weak areas in the ground, and implementing countermeasures such as ground improvement or using lightweight embankment materials in those zones.

## Introduction

 Ogata Village, Minami Akita District, Akita Prefecture, known as one of the leading agricultural production areas in Japan, has supported food production since the land reclamation project in 1957. However, the degradation of agricultural production bases and facilities is significant. Additionally, the deterioration of water quality and the occurrence of differential settlement of structures, which are problems specific to lowland landfills, are factors hindering the realisation of sustainable agricultural production. There are various causes of differential settlement, such as differences in load and soil properties. A previous study clarified the effect of salt leaching on the mechanical properties of clay and examined the possibility of differential settlement occurring^1,2^. They investigated the effects of salt leaching on the consolidation deformation properties of Hachirogata clay. They showed that leaching changes the orientation of clay particles from a random structure to a parallel arrangement, with non-leached clay containing more intra-pores and leached clay exhibiting more inter-pores. They also summarised that these structural changes lead to decreases in the liquid limit, plastic limit, and plasticity index, as well as increases in settlement amount and delays in consolidation deformation.

Salt leaching in clay layers affects the geotechnical properties of clay. Bjerrum^3^ investigated the factors that cause changes in the geotechnical properties of clay and their effects in Drammen, Norway, where soft clay is prevalent. They reported that after salt leaching, the clay showed an 11% decrease in liquid limit and a 50% reduction in undrained shear strength. Torrance^4^ reported that salt leaching decreases the liquid and plastic limits, which increases the liquidity index and causes quick clay to develop into low-activity clay. Ohtsubo et al.^5^ explored the geotechnical, chemical, and mineralogical properties of Ariake clay, a soft sediment of alluvial origin, and examined the occurrence of salt leaching and its role in the development of quick clay. They showed that the shear strength in the remoulded condition decreased with decreasing salt concentration, that quick clay was formed, and clarified that there was a correlation between strength and salt concentration. Furthermore, they reported that the development of quick clay could be mineralogically attributed to low-swelling smectites.

Salt leaching is also known to affect the structure of clay. Sridharan and Javadeva^6^, Van Olphen^7^, Komine^8^, and Bayesteh and Mirghasemi^9^ showed that changes in the microstructure and macrostructure of clay can be explained by variations in the thickness of the electric double layer, as described by the Gouy–Chapman diffusion electric double layer theory. Iwata and Kita^10^ described that clay dispersion and aggregation occur depending on the magnitude of the relationship between the repulsive and attractive forces, which are governed by the dynamic potential between negatively charged clay particles, according to the electric double layer concept. They also reported that when clay particles are suspended, their structure becomes densely oriented and impermeable in the dispersed state, and randomly oriented and permeable in the aggregated state. Olsen^11^ suggested that particle structures can be classified into two types: inter-cluster and intra-cluster. Bayesteh and Bayat^12^ reported that, in high-salinity water, particles aggregate and form inter-clusters, whereas in low-salinity water, particles disperse and form intra-clusters. Thus, the ion concentration in the pore water alters the structure of clay particles and affects their physicochemical and mechanical properties. Saadatkhah et al.^13^ measured changes in the geotechnical properties of seafloor clayey silt due to salt leaching using flushing experiments and assess the implications of these changes on the stability of siliciclastic continental margins with 2D limit equilibrium modelling. They concluded a ~ 50% decrease in undrained cohesive strength of seafloor sediment after flushing, as well as a decrease in its shear strength, bulk density, and moisture content, which is similar to that reported for subaerial quick clays undergoing salt leaching. Focused on improvement of the geotechnical properties of remoulded quick clay, Loshelder et al.^14^ studied the mixing of salts recycled from waste incineration fly ash into remoulded quick clay. They found that a change in the geotechnical properties was immediately evident upon the mixing and the effect on the shear strength increased further with increasing storage time. Their findings showed that recycled salt could potentially be used to improve geotechnical properties as a viable, low-emission alternative to cement-based binders.

Based on previous research findings, this study examines the effects of salt leaching on the micromechanical behaviour and structure of Hachirogata clay. Specifically, leached specimens were artificially prepared using boring specimens collected from reclaimed land, and strength tests were conducted on the micro-areas of both non-leached and leached specimens using a micro-indenter. It is noted that the use of micro-indentation tests to investigate the effect of salt leaching on the strength of clay is novel.

## Materials and methods

This study used a bore sample (undisturbed sample) collected from a depth of 5.0–5.9 m during a geological survey of the irrigation channel in Hachirogata. The physical properties of the Hachirogata clay are shown in Table 1. Using this sample, a leached specimen was artificially prepared^1,2^.

**Table 1:** Physical of properties of Hachirogata clay.

Liquid limit	Plastic limit	Plasticity index	Natural water content	Particle density	Particle size composition			Depth
*w*_L_ (%)	*w*_P_ (%)	*I*_P_	*w*_n_ (%)	*r*_s_ (g/cm^3^)	Clay (%) < 2 μm	Silt (%) 2 μm to 75 μm	Sand (%) 75 μm to 2 mm	m
218.7	64.4	154.3	210.3	2.390	35.0	64.5	0.5	5.00-5.89

### Salt-leached sample under remoulded conditions

The bore samples and distilled water were mixed in a mass ratio of 1:2, and the mixture was thoroughly stirred. The mixture was poured onto a dialysis membrane and placed in distilled water for a specified period, during which the salinity of the clay was leached by osmotic pressure. The degree of leaching was confirmed by measuring the electrical conductivity (EC) of the surrounding distilled water. While the mixture was allowed to stand, the following precautions were taken to promote leaching: (1) EC values were regularly measured during the leaching period, and the dialysis membrane sample was inverted after each measurement; (2) the distilled water was replaced when the EC value stabilised. In this study, the leached samples were prepared over a standing period of approximately four weeks (hereinafter referred to as 4 W-leached).

**Fig. 1:**
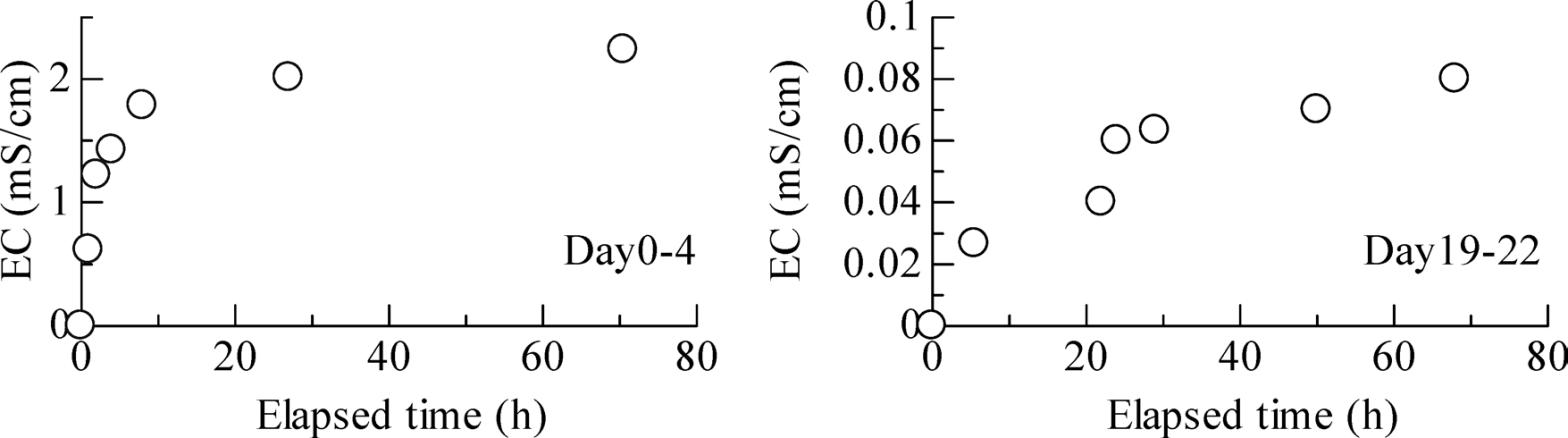
Change in the electrical conductivity (EC) of distilled water during the preparation of the remoulded leached samples.

**Fig. 2:**
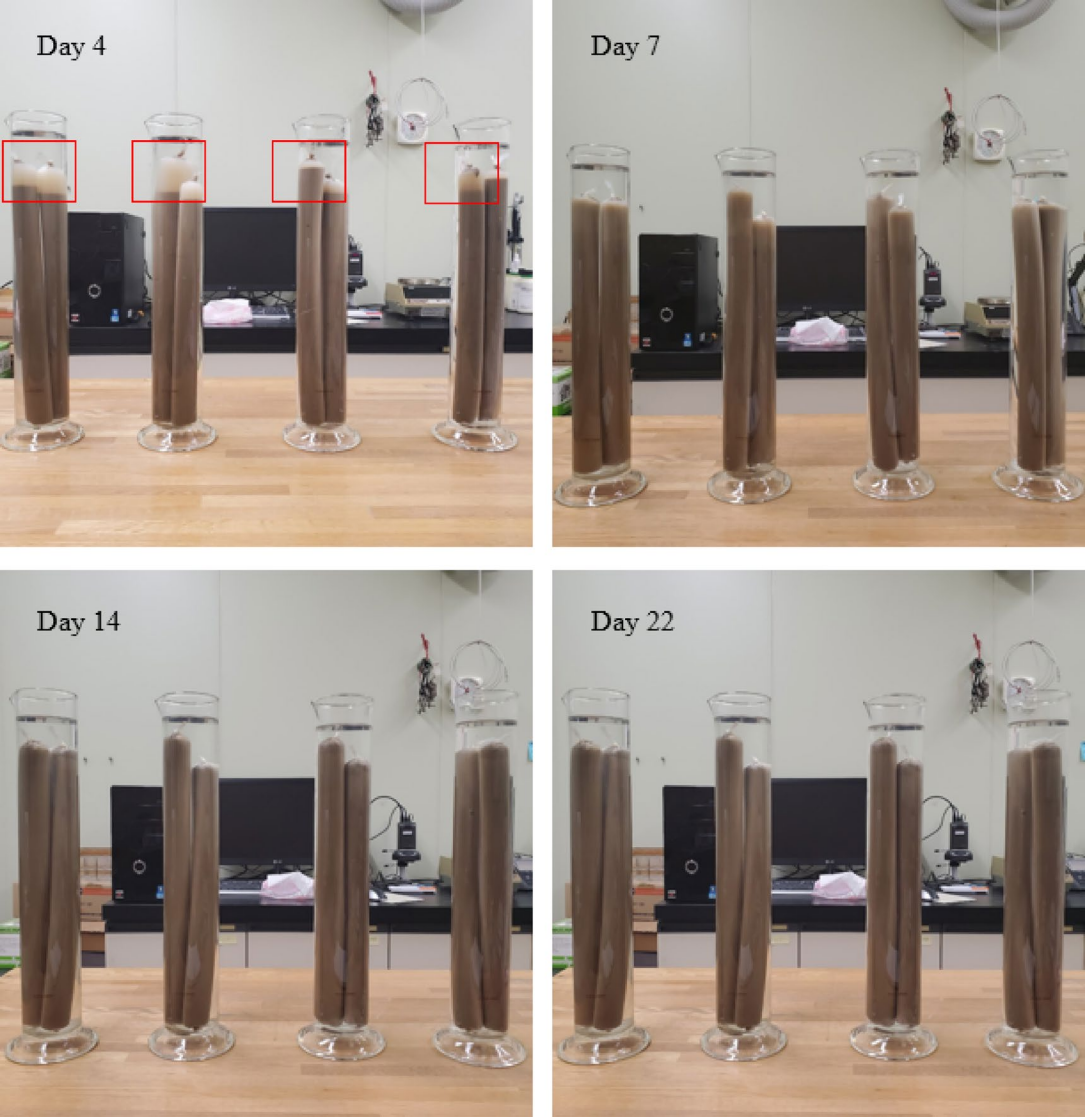
Images showing differences in the settling behaviour of the samples during artificial leaching.

Figures 1 and 2 show graphs of the changes in EC values of the surrounding distilled water and the leached samples during settling. As shown in Fig. 1, the initial EC values rapidly increased until 15 h, after which they remained nearly constant. When the water was changed, the EC values gradually increased but remained relatively low. Comparing the EC values from days 0 to 4 and 19 to 22 after leaching, it was found that the rate of change in EC values was smaller during days 19 to 22. Furthermore, based on the state of the samples during standing (the red square in Fig. 2), while a distinct boundary between the clay particle suspension and the solution could be observed at the top of the sample on the fourth day of settling, this boundary dissipated as the settling period increased. Thus, it was visually confirmed that the sample in the dialysis membrane flocculated on day four but gradually changed to a dispersed state.

### Salt-leached sample under undisturbed conditions

Two types of undisturbed leaching samples were prepared: a block sample (hereinafter referred to as B-leached), consisting of a 20 mm-thick cut from a boring sample with a diameter of 76 mm, and a piece sample (hereinafter referred to as P-leached), consisting of approximately six equal parts of this block sample. This was done to prepare samples with various EC values in a short period, especially to prepare samples with low EC values by using small sample pieces. The B-leached sample was prepared by wrapping the dialysis membrane directly around the clay sample, whereas the P-leached sample was prepared by adding the clay sample and distilled water to the dialysis membrane. These samples were then immersed in distilled water and leached in the same manner as the remoulded leaching samples. The leaching period varied by sample, ranging from 3 to 45 d.

**Fig. 3:**
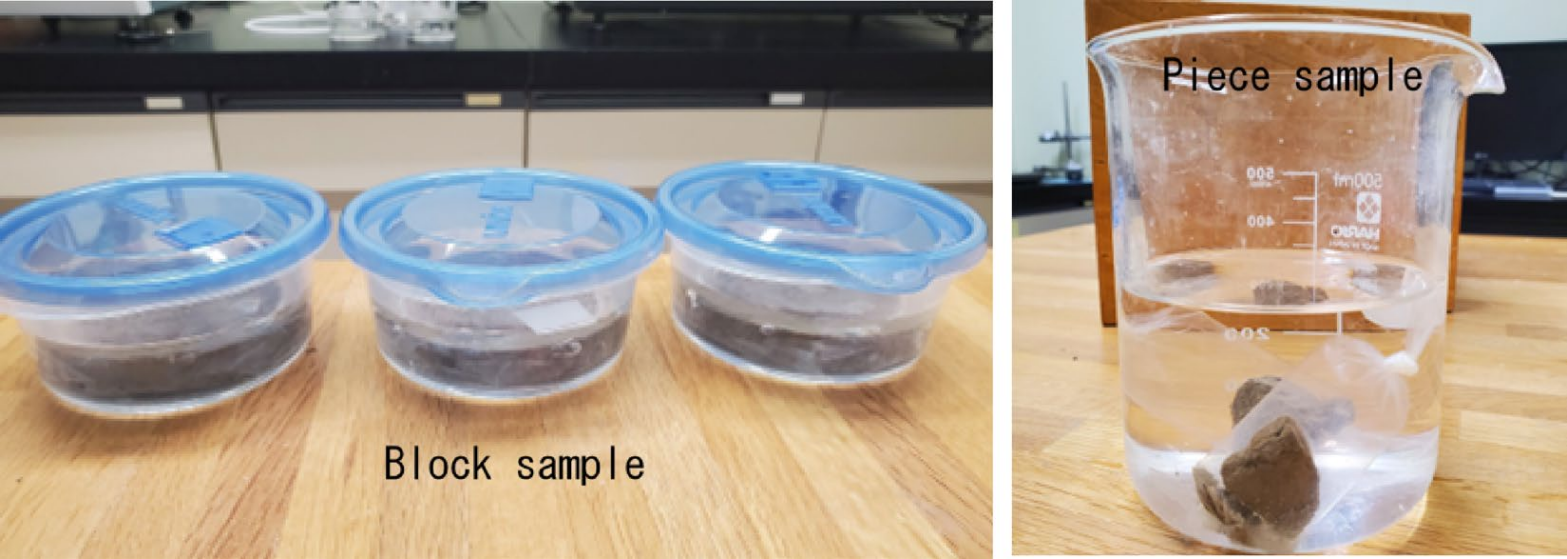
Images showing the leaching method and conditions during the preparation of the undisturbed samples.

**Fig. 4:**
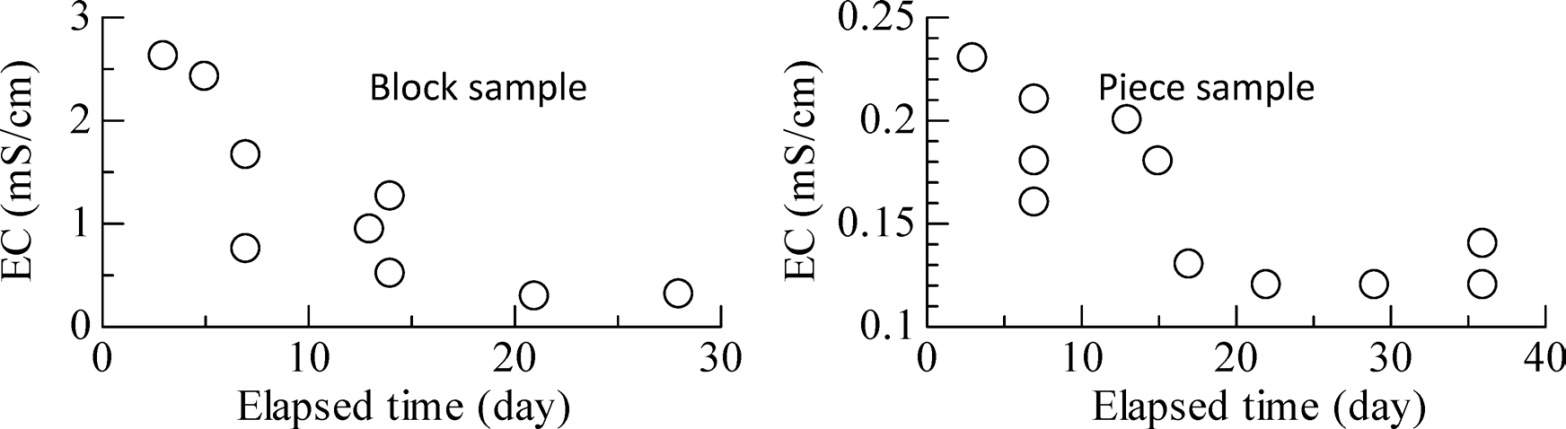
Plots showing changes in the EC of block and piece samples under the influence of osmotic pressure.

The EC was measured by inserting an EC meter directly into the sample after completion of the strength test. Figure 3 shows the state of leaching, and Fig. 4 presents the EC values of the B-leached and P-leached samples. The EC of the sample without leaching was 8.14 (mS/cm). In both samples, a decrease in EC was observed over time. Comparing the respective EC values, after 3 d, the EC of the B-leached sample was 2.62 (mS/cm), while that of the P-leached sample was 0.23 (mS/cm), indicating a significant initial decrease in the EC value of the piece sample. Since the EC value of the undisturbed sample decreased, the leaching method using the dialysis membrane was also effective for the undisturbed sample.

### Micro-indentation testing method

When the leaching method adopted in this study was applied to clay samples, leaching occurred more actively at the surface of the clay sample, that is, where it was in contact with the dialysis membrane. Consequently, leaching heterogeneity within the sample could be expected. This heterogeneity leads to variations in soil strength measured by conventional strength tests, and it is believed that the effects of leaching on soil strength cannot be accurately evaluated. Therefore, this study used the micro-indentation testing method^15,16^ to investigate changes in the microscopic mechanical properties of the soil owing to salt leaching. Their research has examined the *c* value based upon the single loading test showed the almost same value as the *q*_u_/2 value derived from unconfined compression test.

**Fig. 5:**
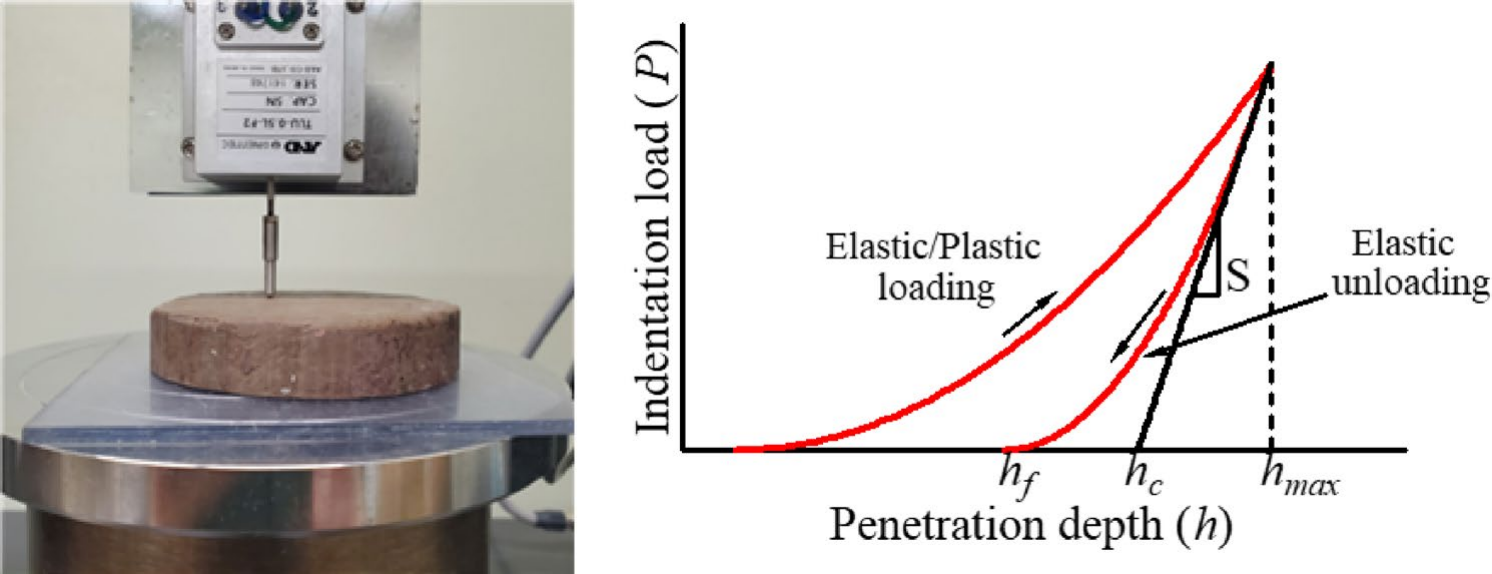
Image of the indentation test setup and the parameters derived from the indentation load–depth curve.

In this study, measurements were performed using a universal compression tester (A&D Co., Ltd., RTG-1210). Hereafter, this is referred to as a micro-indenter. The main features of this tester include a load cell capacity of 5 N, a load measurement range of up to 1/500 of the load cell capacity, a load accuracy of ± 1.0% of the reading, a speed range of 0.05–1000 mm/min, and a speed accuracy of 0.01 mm/min. The tip of the indenting rod was spherical with a diameter of 3 mm. Using this micro-indenter, the indentation depth (h) and indentation load (P) were measured in one loading cycle at a loading speed of 0.1 (mm/s), with an indentation depth of 1.0 (mm). Figure 5 shows the test setup and the parameters derived from the indentation load–depth curve. Accordingly, the hardness and Young’s modulus of the materials could be calculated.

Hardness is the most basic property that can be measured through indentation testing. Its calculation requires three parameters: the load *P*, the contact area *A*_*c*_, and the contact stiffness *S* between the indenter and the sample. The contact stiffness *S* corresponds to the slope at the initial point of the unloading curve in the load–displacement curve (Fig. 5).

1 $$S = \frac{{dP}}{{dh}}\left| {_{{P_{{\max }} }} } \right.$$

The contact depth *h*_*c*_ is typically shallower than the maximum indentation depth *h*_*max*_ due to the elastic deformation of the surface surrounding the contact point.

2 $$h_{c} = h_{{\max }} - \varepsilon \frac{P}{S}$$

where *ε* is a constant that depends on the shape of the indenter: it is 0.75 for a spherical or triangular pyramidal indenter, and 0.72 for a circular indenter, within the range of small indentation depths. The contact area *A*_*c*_ between the indenter and the sample was geometrically calculated using the radius *r* of the spherical tip.

3 $$A_{c} = 2\pi rh_{c}$$

From the above, the hardness *H* can be calculated using the following formula:

4 $$H = \frac{P}{{A_{c} }}$$

As this method considers the effect of surface elastic deformation in the calculation of the area, the resulting hardness reflects only the plastic properties of the material. Regarding Young’s modulus, the modulus obtained from the load–displacement curve is the composite elastic modulus *E*_*r*_ of the indenter–sample system and is calculated using the following equation:

5 $$\:\text{}\mathrm{S}\mathrm{=\:}\frac{\mathrm{2}}{\sqrt{\pi}}{\mathrm{E}}_{\mathrm{r}}\sqrt{{\mathrm{A}}_{\mathrm{c}}}$$

**Fig. 6:**
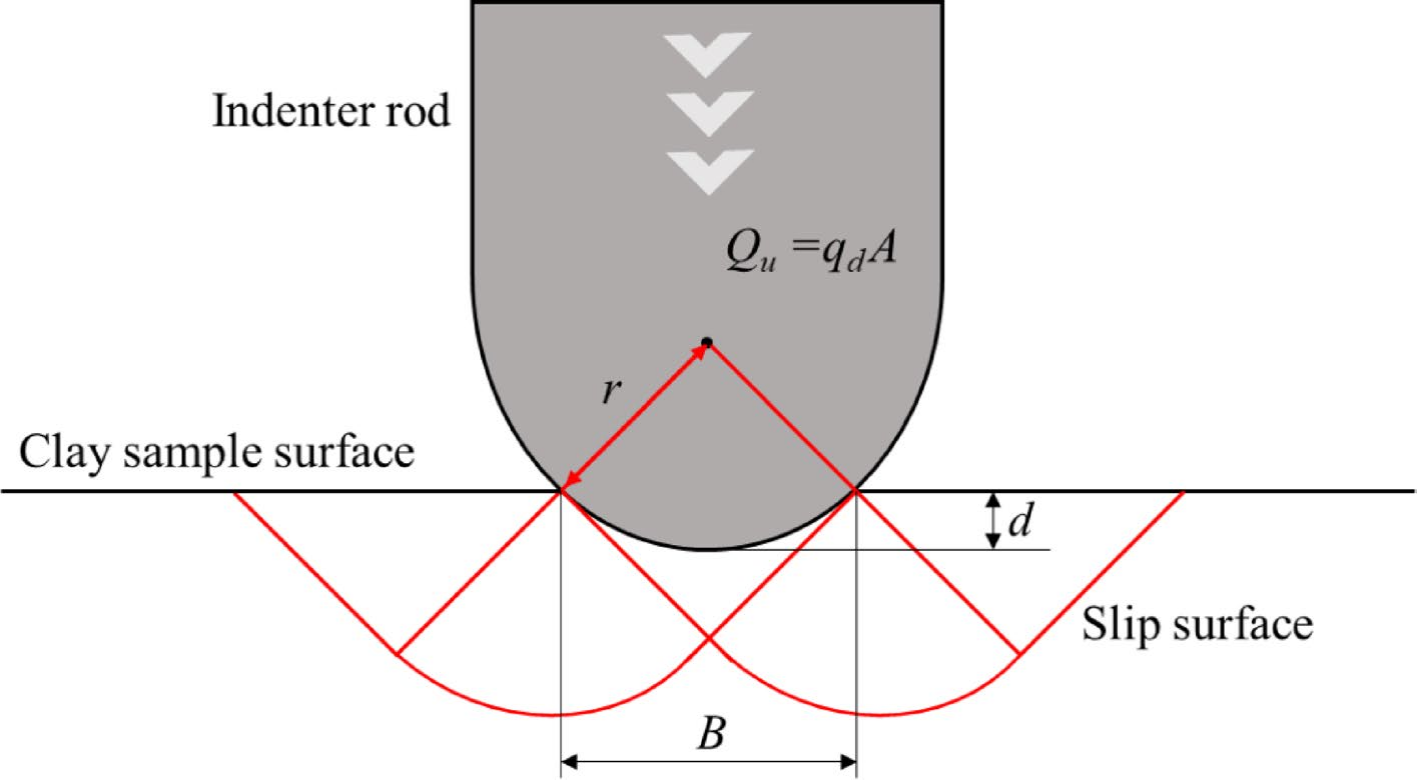
Schematic of clay surface failure caused by an indenting rod.

The microscopic strengths of the clay samples were obtained using the following method: Terzaghi derived the ultimate bearing capacity formula for the general shear failure of shallow continuous foundations by modifying the plastic solution proposed by Prandtl to determine the bearing capacity of metallic materials. Assuming that the sample surface fails owing to the indentation of the indented sphere, as shown in Fig. 6, Terzaghi’s Eq. (6) can be applied to calculate the bearing force:

6 $$Q_{u} = q_{d} A = \tfrac{{\pi B^{2} }}{4}\left( {1.3cN_{c} + 0.3\gamma BN_{\gamma } } \right)$$

where *q*_d_ is the ultimate bearing capacity of the soil, *B* is the contact width, *c* is the cohesion, *γ* is the unit weight of the soil, and *N*_*c*_ and *N*_*γ*_ are the bearing capacity coefficients. When the contact area is projected onto a plane, the contact width *B*, corresponding to a penetration depth *d* of the indented sphere, is given by $\:B=2\sqrt{2dr-{d}^{2}}\le\:2r$. When the internal friction angle is *ϕ* = 0, the bearing force coefficients *N*_*c*_ and *N*_*γ*_ are 5.14 and 0, respectively, and the cohesion *c* is expressed as follows:

7 $$c = \frac{{Q_{u} }}{{21d\left( {2r - d} \right)}}$$

This study used Terzaghi’s bearing capacity formula and the geometrical conditions described above to convert the micro-indenter test results into cohesive strength.

## Results and discussion

### Micro-indentation test results for the remoulded sample

**Fig. 7:**
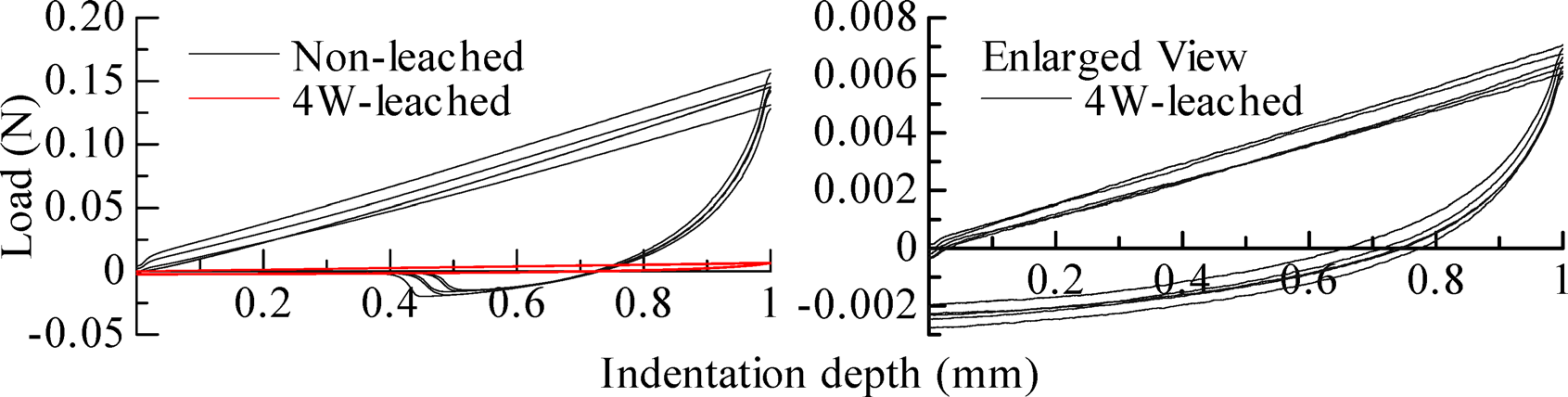
Relationship between load and indentation depth for the remoulded samples.

A micro-indentation test was conducted on the 4 W-leached and non-leached specimens under remoulded conditions. The specimens were prepared by filling the remoulded sample into a Petri dish with a capacity of approximately 14.7 cm^3^ (Diameter: 4.32 cm, Height: 1 cm). Indentation was performed five times for each specimen. Figure 7 shows the load–displacement curves, and Table 2 lists the hardness *H*, composite elastic modulus *E*_*r*_, cohesion *c*, EC, and water content *w* of each sample. The water content of both samples was adjusted to be approximately the same.

Figure 7 shows that the shape of the load–displacement curve for the 4 W-leached specimen was significantly different from that of the non-leached specimen, with a considerably lower maximum load value. As shown in Table 2, when the EC after leaching decreased to one-eighteenth of its pre-leaching value, all measured indices also decreased: *H* to one-twenty-second, *E*_*r*_ to one-twenty-fourth, and *c* to one-twenty-second of their respective pre-leaching values. This result indicates that the strength and stiffness of the remoulded clay sample decreased significantly after leaching. This reduction is attributed to leaching, which decreased the number of ions in the clay, causing its structure to disperse. This dispersion weakened the binding forces between particles and subsequently lowered the load response of the leached sample.

**Table 2:** Parameters of the remoulded specimens obtained from micro-indentation tests.

Sample	Electrical conductivity	Water content	Hardness	Composite modulus	Cohesion
(Num.)	*EC* (ms/cm)	*w* (%)	*H* (N/mm^2^)	*E*_r_ (N/mm^2^)	c (kN/m^2^)
Non-leached (5)	8.60	185.0	0.017	0.45	3.68
Leached (5)	0.48	187.4	0.00078	0.019	0.17

### Micro-indentation test results for the undisturbed sample

**Fig. 8:**
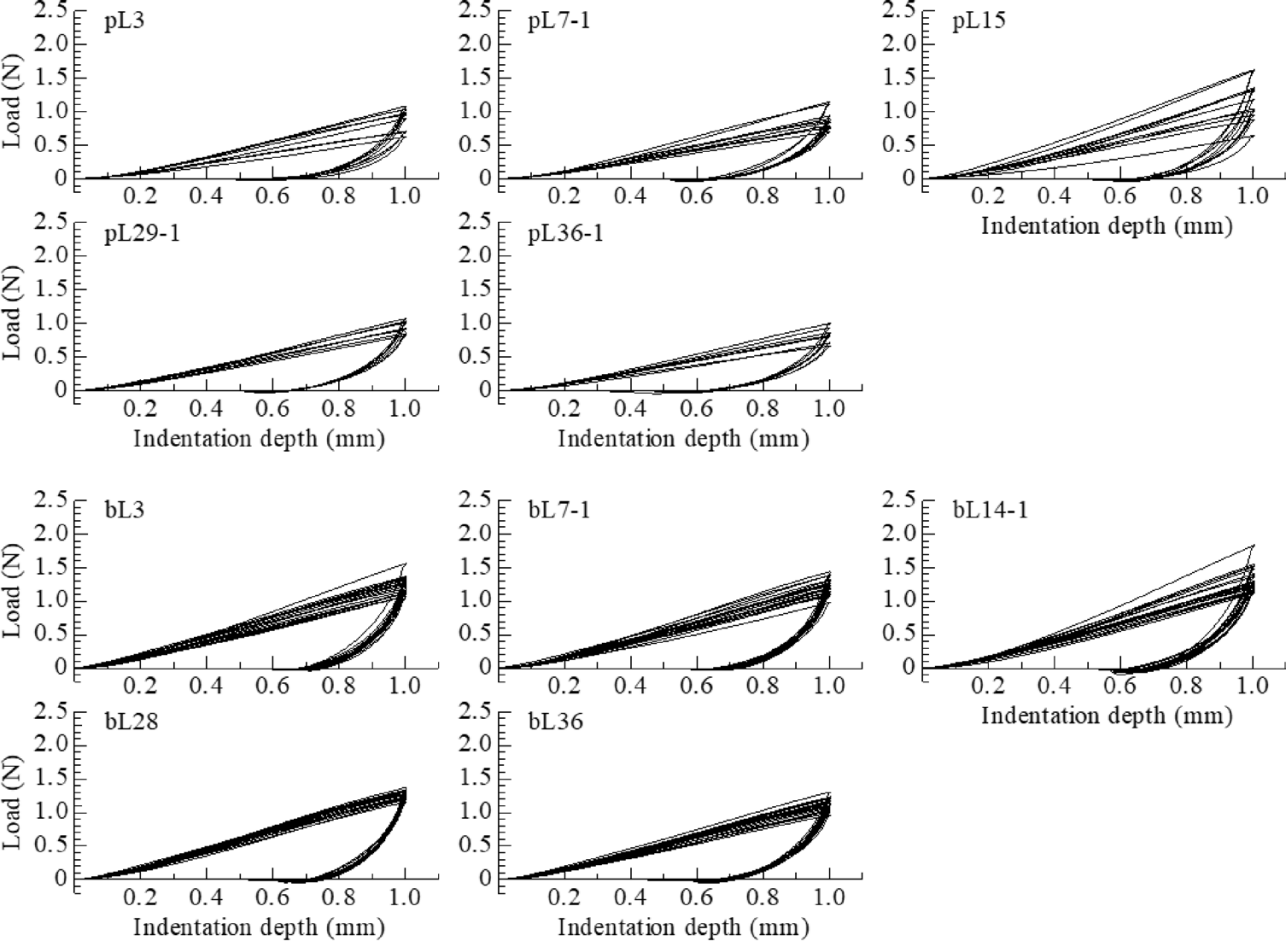
Relationship between load and indentation depth for the undisturbed samples.

From the load–displacement curves shown in Fig. 8, it was confirmed that the maximum load of both samples decreased with leaching time. Furthermore, the maximum load values of P-leached and B-leached samples were lower value than those of B-leached samples. In addition, for both samples, the variability in test results decreased as the leaching period progressed. According to these results, although leaching can be considered a weathering phenomenon in the soil, its progression tends to homogenise the soil’s locally complex structure. In other words, there is a strong correlation between the elimination of heterogeneous soil structures due to leaching and the deterioration of load response characteristics. Therefore, it is necessary to simultaneously observe the soil structure to collect further data to deepen the analysis.

As shown in Table 3, the EC value decreased with leaching time in both samples. In the P-leached sample, despite some variation, the cohesion *c* decreased as EC decreased. In contrast, in the B-leached sample, no significant change in *c* was observed until the 21st day of leaching, followed by a substantial decrease after the 28th day. The hardness *H* was 0.08–0.15 (N/mm^2^) for the P-leached samples and 0.12–0.18 (N/mm^2^) for the B-leached samples, showing no significant difference between the samples. The composite elastic modulus *E*_*r*_ ranged from 1.30 to 2.74 (N/mm^2^) for the P-leached samples and from 2.87 to 4.48 (N/mm^2^) for the B-leached samples, with the latter exhibiting higher values. The water content *w* ranged from 200.3% to 220.5% for P-leached samples and from 188.0 to 220.7% for B-leached samples.

**Table 3:** Parameters of the undisturbed specimens obtained from micro-indentation tests.

	Sample	Electrical conductivity	Water content	Leaching period	Hardness	Composite modulus	Cohesion
	(Num.)	EC (ms/cm)	w (%)	(day)	H (*N*/mm^2^)	E_*r*_ (*N*/mm^2^)	c (kN/m^2^)
P-leached	pL3 (10) pL7-1 (10) pL7-2 (10) pL7-3 (10) pL7-4 (10) pL13 (10) pL15 (10) pL17 (10) pL22 (10) pL29-1 (10) pL29-2 (10) pL36-1 (10) pL36-2 (10)	0.23 0.21 0.16 0.18 0.21 0.20 0.18 0.13 0.12 0.12 0.12 0.12 0.14	204.7 200.3 208.6 214.3 201.5 214.2 210.8 210.9 211.8 215.8 210.4 215.3 220.5	3 7 7 7 7 13 15 17 22 29 29 36 36	0.11 0.13 0.11 0.08 0.11 0.13 0.15 0.11 0.12 0.12 0.10 0.10 0.11	2.13 2.47 2.08 1.30 1.83 2.74 3.23 2.12 2.37 2.29 2.02 2.02 2.33	22.4 26.9 22.8 15.8 23.5 26.6 31.3 23.0 25.8 24.5 21.9 21.3 23.6
B-leached	U-Nleached (20) bL3 (20) bL5 (20) bL7-1 (20) bL7-2 (20) bL13 (20) bL14-1 (20) bL14-2 (20) bL21 (20) bL28 (20) bL36 (20) bL41 (20) bL45 (20)	8.14 2.62 2.42 0.75 1.66 0.94 1.26 0.51 0.29 0.31 0.30 0.31 0.29	188.0 189.4 218.3 188.7 211.0 193.9 217.9 204.3 200.4 220.7 211.4 201.3 201.9	0 3 5 7 7 13 14 14 21 28 36 41 45	0.17 0.15 0.18 0.15 0.17 0.16 0.16 0.17 0.17 0.15 0.14 0.13 0.12	3.37 3.36 4.00 3.22 3.80 3.73 3.25 4.00 4.48 3.49 2.87 3.01 2.55	36.0 31.5 37.4 30.9 35.4 33.9 33.9 36.2 37.1 32.2 28.9 28.7 26.2

Focusing on the relationships between logarithmic EC and *c*, and between *w* and *c*, the simple correlation coefficient *r* was 0.74 for logarithmic EC–*c* and − 0.29 for *w*–*c*, indicating a strong positive correlation between logarithmic EC and *c*. In addition, multiple regression analysis was conducted using *c* as the objective variable and EC and *w* as explanatory variables to examine their effects on cohesion. Table 4 presents the results of this analysis. The *R*-squared value adjusted for degrees of freedom *R*^2^ was 0.74. Since this falls within the 0.6–0.8 range, which is considered significant, it indicates that the explanatory variables *w* and EC can adequately explain the objective variable *c*.

The significance *F* of the estimated regression equation was 0.0001, which is less than 0.05, indicating that a useful regression equation was obtained. Regarding the *t* value, which indicates the degree of influence of the explanatory variables, the value of the EC was 4.85, while that of *w* was 0.47, indicating that EC has a stronger influence on the objective variable. The *P*-value, which indicates the significance of the estimated coefficients, was 0.00007 for EC and 0.64 for *w*, suggesting that EC *i*s more closely related to *c* than *w*. Finally, the coefficient representing the magnitude of each variable’s effect, after controlling for the influence of other variables, was 9.11 for EC and 0.05 for *w*, indicating that EC has a substantially greater effect. Since the significance of EC was confirmed for all parameters, it was statistically determined that EC has a stronger effect on *c* than *w* in structured, undisturbed clay.

**Table 4:** Results of the multiple regression analysis.

Parameter	Value
Adjusted *R*^2^	0.74
Significance *F*	0.0001
*t*-value (*EC*)	4.85
*t*-value (*w*)	0.47
*P*-value (*EC*)	0.00007
*P*-value (*w*)	0.64
Coefficient (*EC*)	9.11
Coefficient (*w*)	0.05

**Fig. 9:**
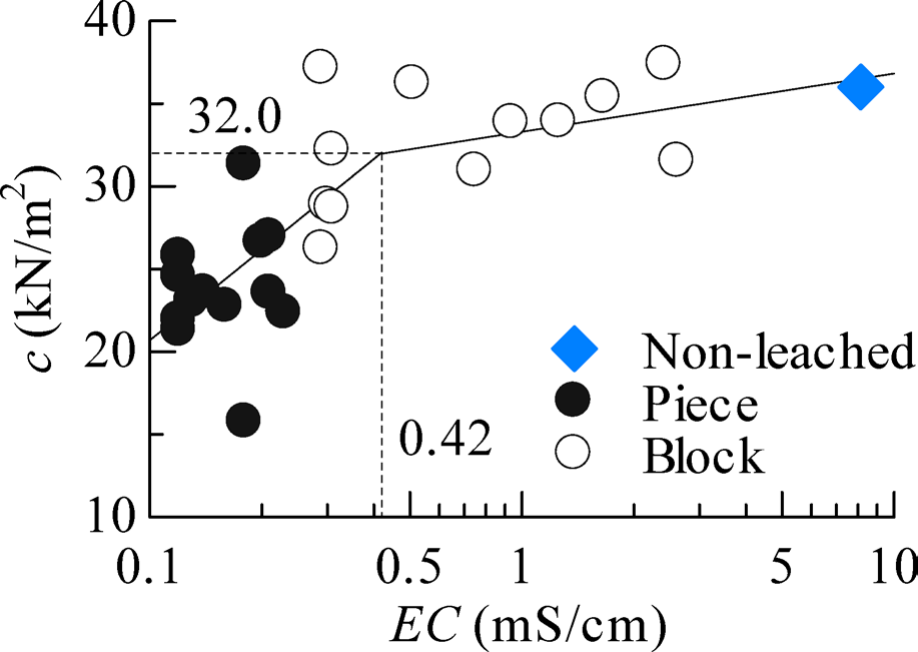
Cohesion as a function of EC and determination of the threshold EC.

Furthermore, a review of the EC–*c* relationship in Fig. 9 showed that the *c* value decreased significantly after a certain EC threshold. As shown in Table 3, strength did not change significantly until the leaching period exceeded approximately four weeks, then began to decrease after 30 d. Therefore, considering that the EC decreases with the progression of the leaching period, it can be inferred that the EC value at approximately 30 d of the leaching period marks a transition point associated with a significant reduction in strength.

To calculate the EC at which the strength dropped sharply, all test results were divided into two intervals such that both intervals showed high correlation coefficients. When calculating the intersection points of each regression equation, the EC was 0.42 (mS/cm), and the *c* was 32.0 (kN/m^2^). It has been believed that a decrease in strength due to salt leaching occurs only when the sample is disturbed, and that strength does not decrease when the sample is undisturbed. However, from a microscopic perspective, this study experimentally demonstrated that the strength of clay remains roughly constant at 30–35 kN/m^2^ until the EC decreases to 0.42 mS/cm, after which the strength rapidly decreases when the EC falls below 0.42 mS/cm.

Since the amount of ions in clay affects the attractive and repulsive forces acting between clay particles, it is believed that the binding forces between particles decrease as salt leaching occurs and ion content declines. In other words, when a load is applied to clay maintaining the threshold EC value, the clay structure is maintained; however, when a load is applied to clay with an EC value below the threshold, the clay deforms easily due to a decrease in binding force. Therefore, the threshold EC value is expected to serve as an indicator of clay weakening.

### Relationship between the microscopic structure and micromechanical properties of Hachirogata clay

**Fig. 10:**
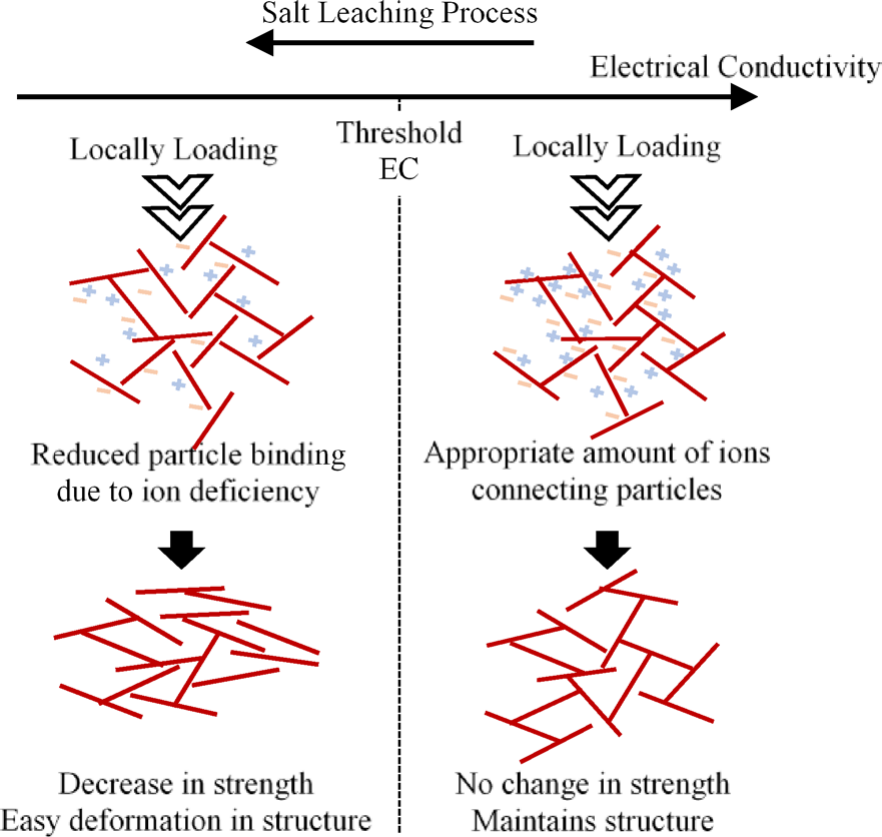
Schematic representation of the effects of changes in ion content on the microscopic strength properties of clay.

Takahashi and Kanayama^1,2^ showed that changes in the Hachirogata clay structure caused by leaching affect the clay’s consistency limit and consolidation deformation characteristics. In addition to the above results, this study discusses the strength properties obtained from micro-indentation tests and the clay’s microscopic structure. Figure 10 shows a schematic of the effects of changes in ion content on the microscopic strength properties of clay, considering the results of this study. Because the ion content in clay affects the attractive and repulsive forces acting between clay particles, salt leaching is considered to occur, and the ion content in clay decreases, which reduces the binding force between clay particles.

When a load is applied to clay that retains an appropriate ion content, the clay structure is maintained and exhibits sufficient resistance to the load; this corresponds to the behaviour of specimens with an EC of 0.42 or higher in the micro-indentation results. Conversely, when a load acts on clay with ions eluted due to leaching, the clay structure is not maintained due to the decreased binding force between particles, resulting in insufficient resistance to the load; this corresponds to the behaviour of the specimen with an EC of 0.42 or less in the micro-indentation results. Therefore, a stepwise correlation was confirmed between EC and the micromechanical properties of the structured, undisturbed clay.

In the relationship between EC and cohesion, cohesion suddenly decreases when EC falls below the threshold value, which is believed to be related to the diffuse electric double layer^17^. Soil particles carry an electric charge, and near their surface, a Stern layer forms where ions of opposite charge are adsorbed. Outside this layer is the Gouy–Chapman layer, where a concentration gradient forms as ions diffuse due to electrostatic attraction. These two layers together form an electric double layer. It is believed that leaching occurs first in the outer Gouy–Chapman layer and then in the Stern layer closer to the particle surface. Assuming that leaching in the Stern layer, which is closer to the particle surface than the Gouy–Chapman layer, has a greater effect on strength, it can be inferred that strength suddenly decreases when the threshold EC value is exceeded. Note that the threshold EC value is believed to differ depending on the clay mineral; therefore, more data are needed to examine the relationship between EC and strength in greater detail.

## Conclusion

This study aims to elucidate the effects of salt leaching on the physicochemical and micromechanical properties of highly active Hachirogata clay. Leached samples were artificially prepared from bore samples collected at the Hachirogata reclaimed land, and micro-indentation tests were performed on both non-leached and leached samples.

The test results showed that the EC and cohesion of the Hachirogata clay are highly correlated, and it was confirmed that strength decreases rapidly when EC falls below a certain threshold. Therefore, it was clarified that salt leaching decreases the cohesion of clay particles by reducing the number of ions in the clay, making the clay more susceptible to deformation under load. From the results of this study, it was inferred that local salt leaching, which alters the micromechanical behaviour of clay, is one of the factors contributing to ground differential settlement or slip failure. It is expected that such ground-related problems can be mitigated by identifying locations with low EC values, that is, weak areas in the ground, and implementing countermeasures such as ground improvement or the use of lightweight embankment materials in those areas.

## Data Availability

The datasets generated and/or analysed during the present study are available from the corresponding author upon reasonable request.
